# Predictors of Metastatic Lymph Node Burden in Colorectal Cancer: A Negative Binomial Regression Analysis

**DOI:** 10.3390/life16091507

**Published:** 2026-09-09

**Authors:** Laurențiu Augustus Barbu, Stelian-Stefaniță Mogoantă, Tiberiu Stefăniță Țenea Cojan, Nicolae-Dragoș Mărgăritescu, Cecil Sorin Mirea, Marius Cristian Marinaș, Liviu Vasile

**Affiliations:** 1 Department of Surgery, Railway Clinical Hospital Craiova, University of Medicine and Pharmacy of Craiova, 2 Petru Rares Street, 200349 Craiova, Romania; laurentiu.barbu@umfcv.ro (L.A.B.); tiberiu.tenea@umfcv.ro (T.S.Ț.C.); 2 Department of Surgery, Emergency County Hospital, University of Medicine and Pharmacy of Craiova, 2 Petru Rares Street, 200349 Craiova, Romania; ssmogo@yahoo.com (S.-S.M.); dmargaritescu@yahoo.com (N.-D.M.); mirea_cecil@yahoo.com (C.S.M.); vliviu777@yahoo.com (L.V.); 3 Department of Human Anatomy, University of Medicine and Pharmacy of Craiova, 2 Petru Rares Street, 200349 Craiova, Romania

**Keywords:** colorectal cancer, prognostic nutritional index (PNI), lymph node metastasis, immunonutrition, inflammatory biomarkers, negative binomial regression

## Abstract

Background: The prognostic value of preoperative inflammatory and nutritional biomarkers in colorectal cancer has been extensively investigated, but their association with metastatic lymph node burden remains insufficiently explored. This study evaluated whether routinely available biomarkers are associated with the number of metastatic lymph nodes in patients undergoing colorectal cancer resection. Methods: We retrospectively analyzed 935 consecutive patients who underwent colorectal cancer surgery between 2020 and 2024. Preoperative hemoglobin, neutrophil-to-lymphocyte ratio (NLR), platelet-to-lymphocyte ratio (PLR), and Prognostic Nutritional Index (PNI) were assessed. Negative binomial regression was used to model metastatic lymph node count, with sensitivity analyses accounting for surgical setting and total lymph node yield. Results: In the fully adjusted primary model, tumor location (*p* = 0.021), PNI (adjusted IRR 1.024; *p* = 0.045), mucinous component (adjusted IRR 1.768; *p* = 0.026), and lymphovascular invasion (adjusted IRR 2.197; *p* = 0.012) were associated with metastatic lymph node burden, whereas NLR and PLR were not. After additional adjustment for total lymph node yield, the associations with PNI, mucinous component, and tumor location were attenuated, while lymphovascular invasion remained independently associated with greater nodal burden (adjusted IRR 2.376; *p* = 0.007). Conclusions: Lymphovascular invasion showed the most robust association with metastatic lymph node burden. Although PNI showed a modest association in the primary model, it provided no significant incremental improvement in model fit, while tumor-location associations were sensitive to model specification. These findings require validation in independent cohorts.

## 1. Introduction

Colorectal cancer (CRC) remains one of the leading causes of cancer-related morbidity and mortality worldwide despite advances in screening, surgical treatment, systemic therapy, and multidisciplinary care. Although the tumor–node–metastasis (TNM) classification remains central to prognostic assessment and treatment decision-making, substantial clinical heterogeneity persists among patients with similar pathological stages, supporting the investigation of complementary biomarkers that integrate tumor characteristics with host-related factors [1,2,3].

Systemic inflammation, nutritional status, and host immune competence are increasingly recognized as relevant determinants of CRC progression and prognosis. Accordingly, routinely available inflammatory and immunonutritional indices, including the neutrophil-to-lymphocyte ratio (NLR), platelet-to-lymphocyte ratio (PLR), systemic immune-inflammation index (SII), systemic inflammation response index (SIRI), Controlling Nutritional Status (CONUT) score, and Prognostic Nutritional Index (PNI), have been investigated as inexpensive and accessible prognostic biomarkers [4,5,6,7,8,9,10,11,12]. Among these, PNI, derived from serum albumin concentration and peripheral lymphocyte count, has been extensively evaluated, with previous studies and meta-analyses associating lower PNI with adverse postoperative and long-term oncological outcomes [1,3,7,12,13,14,15,16,17,18]. In addition, nutritional and immunonutritional status may vary throughout the perioperative period, and dynamic changes in these parameters have also been associated with clinical outcomes [16,19,20,21].

However, most previous studies have evaluated inflammatory and immunonutritional biomarkers primarily in relation to postoperative complications, recurrence, disease-free survival, or overall survival [2,12,19]. Studies specifically addressing lymph node involvement have generally treated nodal metastasis as a binary outcome (N0 vs. N+) rather than quantifying the actual number of metastatic lymph nodes [22,23]. Such approaches do not capture the heterogeneity of lymphatic dissemination among node-positive patients and may overlook information contained in metastatic lymph node count. The number of metastatic lymph nodes is inherently a count outcome; therefore, binary logistic regression would reduce nodal involvement to N0 versus N+ and discard information on the extent of nodal dissemination, whereas ordinal approaches would require categorization of the observed counts. Moreover, because metastatic lymph node counts exhibited overdispersion, with the variance exceeding the mean, Poisson regression was not appropriate. Accordingly, negative binomial regression was selected to preserve the quantitative nature of the outcome while accounting for overdispersion.

Therefore, the present study aimed to investigate the association between routinely available preoperative inflammatory and immunonutritional biomarkers and metastatic lymph node burden in patients undergoing colorectal cancer resection. We specifically evaluated NLR, PLR, PNI, and hemoglobin together with relevant clinicopathological characteristics, including tumor location, histological grade, mucinous differentiation, lymphovascular invasion, and perineural invasion, using multivariable negative binomial regression.

## 2. Materials and Methods

### 2.1. Study Design and Patient Selection

This retrospective observational study included consecutive adult patients who underwent emergency or elective oncological colorectal resection for histopathologically confirmed colorectal adenocarcinoma at the Emergency County Clinical Hospital of Craiova, Romania, between January 2020 and December 2024. The Emergency County Clinical Hospital of Craiova is a tertiary university referral center serving both urban and rural populations. All resections were performed according to the oncological principles routinely applied in the institution.

#### 2.1.1. Inclusion Criteria

Patients were eligible for inclusion if they met all of the following criteria:

- Age ≥ 18 years;
- Histopathologically confirmed colorectal adenocarcinoma;
- Emergency or elective colorectal resection;
- Complete clinicopathological data, including histopathological lymph node assessment.

#### 2.1.2. Exclusion Criteria

Patients were excluded if they had:

- Histological diagnoses other than colorectal adenocarcinoma;
- Incomplete laboratory, pathological, or lymph node assessment data.

A complete-case approach was used for the primary analysis. Of 1024 patients initially assessed for eligibility, 89 (8.7%) were excluded because the available retrospective records lacked the minimum laboratory and/or pathological information required for the planned analyses, resulting in a final analytical cohort of 935 patients. Because these excluded cases originated from incomplete historical records and were not retained in a structured analytical dataset, variable-level missingness could not be reliably reconstructed. Some excluded patients may also have had missing information for more than one variable.

### 2.2. Data Collection

Clinical, laboratory, surgical, and pathological data were extracted from electronic medical records, operative reports, and final histopathological reports.

The collected variables included age, sex, preoperative hemoglobin concentration, absolute neutrophil count, absolute lymphocyte count, platelet count, serum albumin concentration, tumor location, tumor grading, the presence of a mucinous component, lymphovascular invasion, perineural invasion, the total number of examined lymph nodes, and the number of metastatic lymph nodes.

Laboratory parameters were obtained from routine preoperative blood tests performed before surgery. All hematological parameters were obtained from the same baseline blood sample and expressed as ×10^3^/μL.

The neutrophil-to-lymphocyte ratio (NLR) was calculated as

NLR = absolute neutrophil count/absolute lymphocyte count

The platelet-to-lymphocyte ratio (PLR) was calculated as

PLR = platelet count/absolute lymphocyte count

The prognostic nutritional index (PNI) was calculated according to the Onodera formula:

PNI = 10 × serum albumin (g/dL) + 0.005 × total lymphocyte count (/mm^3^).

Tumor location was classified as cecum, ascending colon, hepatic flexure, transverse colon, splenic flexure, descending colon, or sigmoid colon. Tumor differentiation was categorized as G1, G1–G2, G2, or G3, while the mucinous component was recorded as present or absent.

### 2.3. Histopathological Assessment

All surgical specimens were processed according to the routine protocol of the Department of Pathology of the Emergency County Clinical Hospital of Craiova.

Histopathological examination included assessment of tumor grade, mucinous component, lymphovascular invasion, perineural invasion, the total number of examined lymph nodes, and the number of metastatic lymph nodes. Tumor location and all histopathological characteristics were obtained from the final pathology reports.

Mucinous differentiation was recorded according to the information available in the final histopathological reports and was analyzed as a binary variable (present vs. absent). Quantitative assessment of the proportion of extracellular mucin was not consistently available in the retrospective records and therefore could not be incorporated into the analysis.

The total number of examined lymph nodes and the number of metastatic lymph nodes were determined from the final pathological examination of the resected surgical specimens performed by pathologists.

The primary outcome of the study was the number of metastatic (positive) lymph nodes.

### 2.4. Statistical Analysis

Data were initially processed using Microsoft Excel 2016 (Microsoft Corp., Redmond, WA, USA) with XLSTAT 2022 (Addinsoft SARL, Paris, France). Statistical analyses were subsequently performed using IBM SPSS Statistics version 27.0 (IBM Corp., Armonk, NY, USA).

Continuous variables were summarized as mean ± standard deviation (SD) or median with interquartile range (IQR), depending on their distribution, whereas categorical variables were expressed as frequencies and percentages. Differences between patients without lymph node metastases (N0) and those with at least one metastatic lymph node (N+) were assessed using the Mann–Whitney U test for continuous variables and the chi-square test for categorical variables.

Because the primary outcome was a count variable, overdispersion was formally assessed by comparing the mean and variance of the metastatic lymph node count, calculating the variance-to-mean ratio and the Pearson dispersion statistic from the Poisson model, and performing an auxiliary overdispersion test. Poisson and negative binomial regression models with identical covariate specifications were additionally compared using log-likelihood, Akaike information criterion (AIC), Bayesian information criterion (BIC), and a likelihood-ratio test. Evidence of substantial overdispersion and superior model fit supported the use of negative binomial regression for the primary analysis.

Univariate negative binomial regression analyses were first performed for the laboratory biomarkers and selected tumor-related characteristics. A fully adjusted multivariable negative binomial regression model was subsequently fitted including age, sex, hemoglobin, NLR, PLR, PNI, tumor grading, tumor location, mucinous component, lymphovascular invasion, perineural invasion, and surgical setting (elective vs. emergency). Given the high proportion of zero metastatic lymph node counts, a zero-inflated negative binomial (ZINB) model was additionally evaluated. The count component of the ZINB model used the same covariate specification as the fully adjusted negative binomial model, whereas the zero-inflation component was specified as an intercept-only logistic model. Zero counts were considered clinically plausible outcomes arising from the same underlying disease process rather than structural zeros generated by a clearly distinct subpopulation. The standard negative binomial and ZINB specifications were compared using log-likelihood, AIC, BIC, and the Vuong test for non-nested models.

Because the number of detected metastatic lymph nodes may depend on the total number of lymph nodes examined, an additional sensitivity analysis was performed by adding total lymph node yield as a covariate to the fully adjusted negative binomial regression model described above. Lymph node yield was modeled as a covariate rather than as an offset because the primary outcome was the absolute number of metastatic lymph nodes rather than a metastatic-node rate relative to the number examined.

Covariate inclusion was based on clinical and biological relevance, data availability, and the objectives of the analysis rather than on statistical significance in the univariate analyses. Additional clinically relevant variables available from the medical and histopathological records were incorporated during model refinement, and surgical setting was included to account for potential confounding related to emergency presentation. All covariates were entered simultaneously, and no automated forward, backward, or stepwise variable-selection procedure was applied. Associations were expressed as incidence rate ratios (IRRs) with corresponding 95% confidence intervals (95% CIs).

Given the retrospective design, no formal a priori power calculation was performed. The cohort included 935 patients, of whom 215 had metastatic lymph nodes. Because the primary outcome was modeled as a count, conventional events-per-variable rules for logistic regression were not directly applicable. Model stability was instead assessed through multicollinearity, residual, influence, and sensitivity analyses.

Model performance was further explored using prediction-error and calibration measures appropriate for the count outcome. Mean absolute error (MAE) and root mean squared error (RMSE) were calculated from the predicted metastatic lymph node counts. Apparent calibration was assessed using the same dataset employed for model fitting. For each patient, the fitted negative binomial model was used to obtain the predicted mean metastatic lymph node count. The calibration slope was estimated by fitting a negative binomial recalibration model with a log link, using the logarithm of the model-predicted mean count as the calibration predictor and a freely estimated intercept. Calibration-in-the-large was assessed separately by fitting a negative binomial model with the logarithm of the predicted mean count included as an offset, such that the estimated intercept quantified systematic under- or overprediction. Model-based 95% confidence intervals were derived from the corresponding regression standard errors. Because these calibration analyses were performed using the same dataset employed for model development, the estimates represent apparent (in-sample) calibration and were not corrected for optimism. No bootstrap resampling or cross-validation was performed. Because conventional ROC AUC and Brier score are primarily designed for binary outcomes, these metrics were additionally calculated in a secondary analysis for the probability of having at least one metastatic lymph node (N+ vs. N0), derived from the fitted negative binomial model.

To assess the incremental contribution of PNI, the fully adjusted negative binomial model was additionally compared with an otherwise identical model excluding PNI using log-likelihood, AIC, BIC, McFadden pseudo-R^2^, and a likelihood-ratio test.

The present analysis was designed to evaluate independent associations with metastatic lymph node burden rather than to develop or validate an individual-level clinical prediction model. Therefore, construction of a predictive nomogram was not prespecified.

Multicollinearity among continuous predictors was evaluated using variance inflation factors (VIFs), with values below 5 considered acceptable.

Additional model diagnostics included estimation of the negative binomial dispersion parameter, examination of Pearson and deviance residuals, and assessment of influential observations using Cook’s distance and leverage diagnostics. Residuals were evaluated for extreme departures and systematic patterns, while observations with Cook’s distance > 1 were considered potentially influential.

To assess the robustness of the primary findings to potentially influential observations, the fully adjusted negative binomial regression model was additionally refitted after excluding the six observations with Cook’s distance > 1. The resulting coefficients, 95% confidence intervals, and statistical significance were compared with those obtained from the primary analysis.

To address potential confounding related to surgical setting, additional sensitivity analyses were performed. Baseline clinical, laboratory, and pathological characteristics were compared between patients undergoing elective and emergency surgery. Continuous variables were compared using the Mann–Whitney U test and categorical variables using the chi-square test. Furthermore, separate negative binomial regression models were fitted for the elective and emergency subgroups to assess the consistency of associations between hemoglobin, NLR, PLR, PNI, and metastatic lymph node burden after adjustment for age and sex.

All statistical tests were two-sided, and a *p*-value < 0.05 was considered statistically significant.

### 2.5. Ethical Considerations

The study was conducted in accordance with the principles of the Declaration of Helsinki and was approved by the Ethics Committee of the Emergency County Clinical Hospital of Craiova (approval no. 2136, 19 September 2025).

Given the retrospective study design and the use of anonymized clinical data, the requirement for informed consent was waived in accordance with institutional regulations. Patient confidentiality was maintained throughout the study by anonymizing all collected data before statistical analysis.

## 3. Results

The baseline characteristics of the study population are summarized in Table 1. A total of 935 patients were included in the analysis. The mean age was 67.9 ± 10.4 years, and 57.6% of the patients were male. The median hemoglobin level was 12.09 g/dL, the median neutrophil-to-lymphocyte ratio (NLR) was 2.93, the median platelet-to-lymphocyte ratio (PLR) was 155.00, and the median Prognostic Nutritional Index (PNI) was 33.00. The sigmoid colon was the most common tumor location (42.9%)**,** most tumors were moderately differentiated (G2, 78.4%), and a mucinous component was identified in 21.7% of cases.

No significant differences were observed between patients with and without lymph node metastases regarding hemoglobin, NLR, PLR, PNI, or tumor location. In contrast, tumor grading and the presence of a mucinous component differed significantly between the two groups (both *p* < 0.001) (Table 2).

The metastatic lymph node count showed substantial overdispersion, with a mean of 1.201 and a variance of 13.698, corresponding to a variance-to-mean ratio of 11.405. The Poisson model yielded a Pearson dispersion statistic of 8.805, and formal overdispersion testing confirmed significant extra-Poisson variation (z = 4.646, *p* < 0.001). Model comparison further favored negative binomial regression over Poisson regression, with substantially lower AIC (2098.824 vs. 4501.232) and BIC (2205.316 vs. 4602.884) and a higher log-likelihood (−1027.412 vs. −2229.616). The likelihood-ratio comparison was also significant (LR χ^2^ = 2404.41, *p* < 0.001), supporting the use of the negative binomial model.

Given the high proportion of zero metastatic lymph node counts (720/935, 77.0%), a ZINB model was additionally evaluated. The standard negative binomial model yielded a log-likelihood of −1034.19, AIC of 2110.39, and BIC of 2212.04, compared with −1029.85, 2103.70, and 2210.19, respectively, for the ZINB model. Although the ZINB model showed slightly improved fit indices, the Vuong test did not demonstrate a significant improvement over the standard negative binomial model (z = 0.978, *p* = 0.328). Therefore, considering the nonsignificant Vuong test and the absence of a clinically distinct structural-zero population, the standard negative binomial model was retained.

Univariate negative binomial regression identified PNI, tumor location, and the presence of a mucinous component as significant predictors of the number of positive lymph nodes (Table 3). Higher PNI values and the presence of a mucinous component were associated with an increased metastatic lymph node burden. Among tumor locations, lesions of the hepatic flexure were associated with a lower number of positive lymph nodes, whereas tumors of the descending colon were associated with a higher nodal burden compared with cecal tumors. Hemoglobin, NLR, PLR, tumor grading, and the remaining tumor locations were not significantly associated with the number of positive lymph nodes.

**Figure 1:**
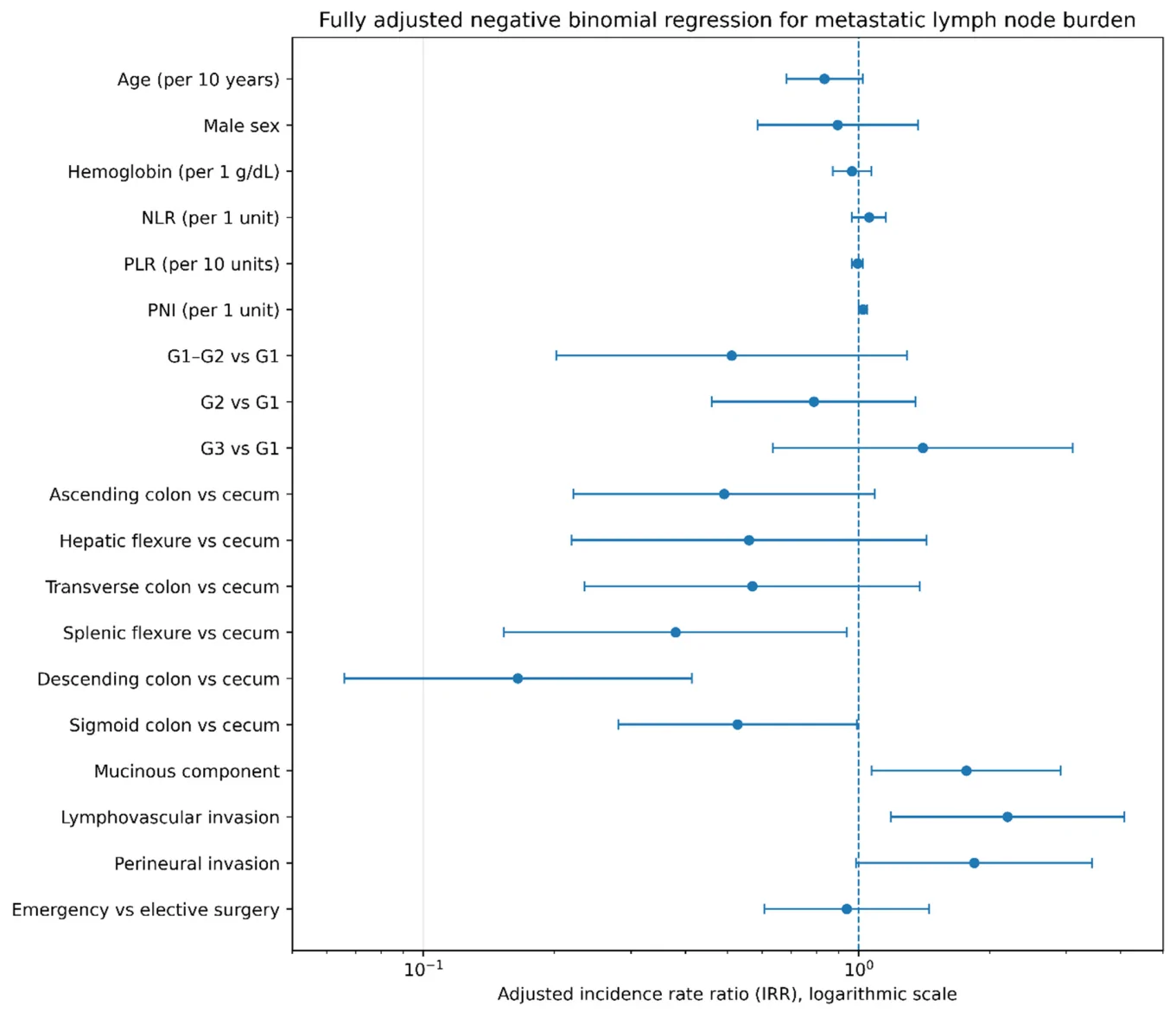
Forest plot of adjusted incidence rate ratios (IRRs) for metastatic lymph node burden. Points and horizontal lines represent adjusted IRRs and 95% confidence intervals, respectively. The dashed line indicates IRR = 1.

In the fully adjusted model, tumor location remained independently associated with metastatic lymph node burden (overall *p* = 0.021). PNI, mucinous component, and lymphovascular invasion were also independently associated with metastatic lymph nodal burden, whereas surgical setting was not (adjusted IRR 0.939, 95% CI 0.608–1.450; *p* = 0.776) (Table 4). The adjusted IRRs and corresponding 95% CIs from the fully adjusted negative binomial regression model are additionally presented graphically in Figure 1.

Notably, the direction of the association for descending-colon tumors differed between the univariate and fully adjusted analyses. This coefficient reversal indicates substantial sensitivity of the location-specific estimate to multivariable adjustment and should therefore not be interpreted as a stable independent protective effect.

In a sensitivity analysis adding total lymph node yield to the fully adjusted model, lymph node yield was independently associated with metastatic lymph node burden (adjusted IRR 1.091, 95% CI 1.062–1.120; *p* < 0.001), while lymphovascular invasion remained independently associated with greater metastatic lymph node burden (adjusted IRR 2.376, 95% CI 1.270–4.445; *p* = 0.007). In contrast, PNI, mucinous component, and tumor location were no longer independently associated with metastatic lymph node burden after adjustment for lymph node yield.

Multicollinearity was assessed among the continuous laboratory and immunonutritional biomarkers included in the multivariable negative binomial regression model. As shown in Table 5, all evaluated biomarkers demonstrated low multicollinearity, with VIF values ranging from 1.01 to 1.17, well below commonly accepted thresholds, indicating no relevant collinearity among these variables.

Emergency patients had significantly higher NLR and PLR values and lower hemoglobin and PNI levels than elective patients (all *p* ≤ 0.002). They also showed a higher metastatic lymph node burden (*p* = 0.004) and a higher frequency of perineural invasion (*p* = 0.013), while tumor grading and anatomical location differed significantly between the two surgical settings (*p* = 0.003 and *p* < 0.001, respectively) (Table 6).

In the stratified sensitivity analysis, neither NLR nor PLR was independently associated with metastatic lymph node burden in either the elective or emergency subgroup. PNI was not significantly associated with metastatic lymph node burden among elective patients but showed a modest independent association in the emergency subgroup (adjusted IRR 1.036, 95% CI 1.003–1.071; *p* = 0.034) (Table 7).

Apparent (in-sample) calibration assessment yielded a calibration intercept of approximately 0.000 (95% CI −0.197 to 0.197) and a calibration slope of 1.000 (95% CI 0.660–1.341). Because calibration was assessed using the same dataset employed for model fitting, these near-ideal point estimates are expected and should not be interpreted as evidence of internal validation or validated calibration. MAE and RMSE were 1.807 and 3.732, respectively. The McFadden pseudo-R^2^ was 0.024, indicating limited explanatory capacity. In the secondary N+ versus N0 analysis, the AUC was 0.662 and the Brier score was 0.168, indicating modest discriminatory performance (Table 8). The addition of PNI did not meaningfully improve overall model performance; changes in log-likelihood (−1034.195 vs. −1034.834), AIC (2110.390 vs. 2109.667), BIC (2212.041 vs. 2206.478), and McFadden pseudo-R^2^ (0.0186 vs. 0.0180) were minimal, and the likelihood-ratio test was not significant (LR χ^2^ = 1.277, *p* = 0.258).

**Figure 2:**
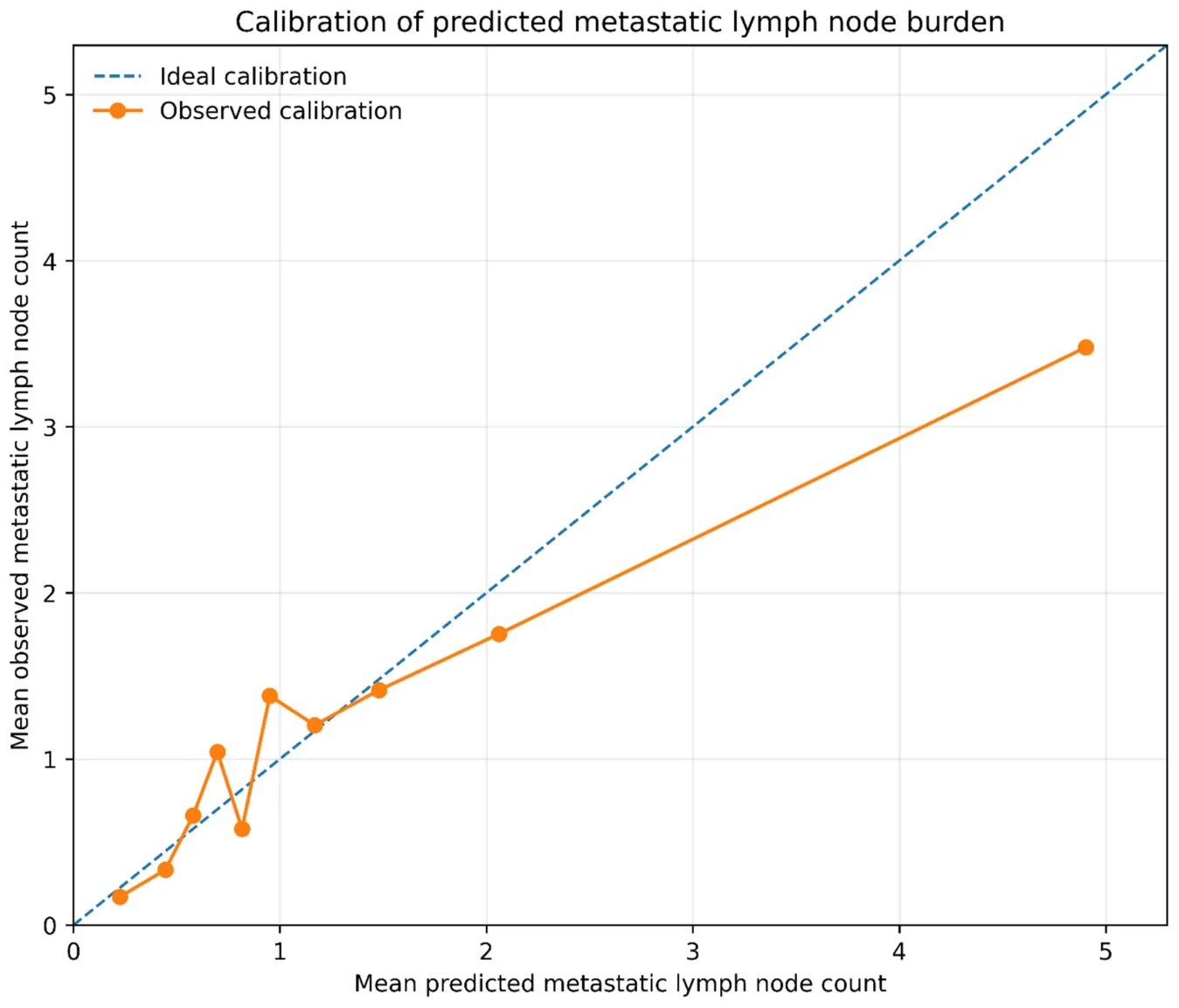
Calibration plot of predicted versus observed metastatic lymph node burden.

The agreement between predicted and observed metastatic lymph node counts is additionally illustrated in Figure 2.

Additional diagnostic assessment supported the negative binomial specification, with an estimated dispersion parameter of 8.343 (95% CI 6.848–9.838). Pearson residuals were centered near zero (mean −0.001; SD 0.994), although 36 observations had absolute Pearson residuals > 2 and 21 exceeded 3. Deviance residuals ranged from −1.024 to 2.650, with only four observations exceeding an absolute value of 2 and none exceeding 3. Influence diagnostics identified six observations with Cook’s distance > 1 (maximum 6.268), indicating the presence of a small number of potentially influential cases.

After exclusion of the six potentially influential observations (*n* = 929), the association between PNI and metastatic lymph node burden was attenuated and no longer statistically significant, whereas lymphovascular invasion remained significant. The main substantive conclusions were therefore unchanged.

Additional stratified analyses showed no significant associations of NLR or PLR with metastatic lymph node burden across tumor-grade or mucinous-status subgroups. PNI was associated with higher nodal burden only in the G1–G2 subgroup (adjusted IRR 1.138, 95% CI 1.022–1.268; *p* = 0.018). However, formal interaction testing showed no significant effect modification by tumor grade, mucinous status, or surgical setting (all interaction *p* > 0.05), although the PNI-by-grade interaction was borderline (*p* = 0.055) (Table 9).

Formal interaction testing showed no significant PNI-by-tumor-location interaction (overall interaction *p* = 0.598). Similarly, no significant interactions were identified between tumor grade and the evaluated biomarkers, although the PNI-by-grade interaction approached statistical significance (*p* = 0.055).

## 4. Discussion

The present study investigated the association between routinely available preoperative inflammatory and nutritional biomarkers and metastatic lymph node burden in a cohort of 935 patients undergoing colorectal cancer resection. In the fully adjusted primary model, tumor location, lymphovascular invasion, mucinous component, and PNI were associated with metastatic lymph node burden, whereas NLR, PLR, tumor grading, perineural invasion, and surgical setting were not. However, after additional adjustment for total lymph node yield, the associations with PNI, mucinous component, and tumor location were attenuated and no longer statistically significant, whereas lymphovascular invasion remained independently associated with greater metastatic lymph node burden. These findings suggest that lymphovascular invasion represented the most robust correlate of metastatic lymph node burden in the present analysis.

Importantly, these associations were identified using negative binomial regression with the number of metastatic lymph nodes analyzed as a quantitative outcome rather than a binary N0 versus N+ variable, allowing a more detailed assessment of nodal disease burden.

The apparent (in-sample) calibration assessment yielded a calibration intercept close to 0 and a slope close to 1. However, because these estimates were obtained from the same dataset used for model fitting, they are inherently optimistic and should not be interpreted as evidence of internal validation or validated calibration. Discrimination for the secondary N+ versus N0 endpoint was modest (AUC 0.662), with a Brier score of 0.168. Accordingly, the present model should primarily be interpreted as an explanatory association model rather than as a clinically validated individual-level prediction tool. Formal internal validation using bootstrap resampling or cross-validation, followed by external validation, would be required before considering its use for individual risk prediction.

The independent association between tumor location and metastatic lymph node burden is biologically plausible and may reflect anatomical, molecular, and microenvironmental heterogeneity across the colorectum. Differences in embryological origin and lymphatic drainage result in distinct regional nodal pathways, with proximal tumors draining predominantly through ileocolic and superior mesenteric lymphatic networks and distal tumors through inferior mesenteric pathways [24,25,26]. Beyond anatomy, right-sided colorectal cancers more frequently exhibit microsatellite instability, *BRAF* mutations, CpG island methylator phenotype, and mucinous differentiation, whereas left-sided tumors are more commonly characterized by chromosomal instability [27,28]. Emerging evidence also indicates substantial spatial variation in the gut microbiota along the colorectum, potentially influencing local inflammation, immune–tumor interactions, and tumor progression [26,28,29]. These interconnected anatomical and biological differences may contribute to location-specific patterns of lymphatic dissemination. However, MSI status, *BRAF* mutations, and microbiome profiles were not available in the present cohort; therefore, these mechanisms remain biologically plausible explanations rather than directly demonstrated mediators of the observed association.

A notable finding was the reversal of the descending-colon coefficient between the univariate and fully adjusted analyses. This change may reflect a suppression effect and/or differences in the distribution of correlated clinicopathological characteristics across anatomical subsites. Therefore, the adjusted estimate for descending-colon location should be considered model-dependent and interpreted cautiously rather than as evidence of an intrinsically protective biological effect.

Importantly, molecular alterations appear to vary along a colorectal continuum rather than according to a simple right–left dichotomy, supporting the biological relevance of evaluating individual anatomical subsites [27].

PNI is a composite biomarker calculated from serum albumin concentration and peripheral lymphocyte count and reflects nutritional reserve, systemic inflammation, hepatic protein synthesis, and cell-mediated immunity. Previous studies have primarily evaluated PNI in relation to postoperative complications, recurrence, treatment tolerance, and survival rather than the absolute number of metastatic lymph nodes [5,30,31].

The relationship between PNI and metastatic lymph node burden should be interpreted within the complex interaction between host immunonutritional status and tumor biology. Because PNI incorporates both serum albumin and peripheral lymphocyte count, it may reflect several overlapping processes, including nutritional reserve, systemic inflammatory activity, and cell-mediated immune competence [4,7,32]. Lymphocytes contribute to antitumor immune surveillance, whereas systemic inflammation and cancer-related metabolic alterations may influence both albumin synthesis and circulating immune-cell populations [32]. However, these host-related factors operate alongside tumor-specific determinants of lymphatic dissemination, including anatomical location, lymphovascular invasion, histological phenotype, and underlying molecular characteristics. Accordingly, the relatively modest association observed for PNI in the present study may indicate that immunonutritional status provides complementary rather than dominant information regarding metastatic nodal burden [1,4,7].

In the present study, PNI was associated with metastatic lymph node burden in the univariate analysis and retained a modest independent association after multivariable adjustment. Each 1-unit increase in PNI was associated with an approximately 2.4% increase in the expected metastatic lymph node count (adjusted IRR 1.024, 95% CI 1.001–1.047; *p* = 0.045). However, this association was confined to emergency patients in the stratified analysis and should therefore be interpreted cautiously. This subgroup-specific finding further suggests that PNI may partly capture acute systemic and immunonutritional perturbations associated with emergency presentation rather than representing a uniform marker of tumor-driven lymphatic dissemination. In the primary model, tumor location, lymphovascular invasion, and mucinous component showed stronger associations with metastatic lymph node burden; however, only lymphovascular invasion remained independently associated after additional adjustment for total lymph node yield.

The absence of an independent association between NLR or PLR and metastatic lymph node burden in the present study should not be interpreted as evidence that these biomarkers lack clinical or prognostic value in colorectal cancer. Rather, our findings are specific to the endpoint investigated—the absolute number of metastatic lymph nodes. PNI retained only a modest association with this endpoint, whereas NLR and PLR were not independently associated with metastatic lymph nodal burden after adjustment for clinicopathological characteristics. These results suggest that systemic inflammatory and immunonutritional biomarkers may have limited ability to reflect the anatomical extent of lymphatic dissemination when compared with tumor-related characteristics.

Importantly, NLR, PLR, and PNI may retain clinical relevance for other outcomes that were not evaluated in the present study. Previous studies have associated systemic inflammatory and immunonutritional status with postoperative morbidity, treatment tolerance, recurrence, disease-free survival, and overall survival in colorectal cancer [5,30,31]. These endpoints reflect broader interactions among tumor biology, systemic inflammation, nutritional reserve, immune competence, treatment response, and postoperative recovery and are therefore biologically distinct from metastatic lymph node count. Consequently, the present findings should not be extrapolated to conclude that NLR, PLR, or PNI lack prognostic utility for postoperative or long-term oncological outcomes.

A recent systematic review and meta-analysis including 43 studies and 19,214 patients demonstrated that low PNI was consistently associated with poorer overall survival in colorectal cancer, although associations with disease-free and progression-free survival were less consistent, suggesting that the prognostic value of PNI depends on the clinical endpoint evaluated [1]. Additional studies have confirmed that PNI independently predicts postoperative complications and survival and improves prognostic accuracy when incorporated into multivariable models [2,7,14]. Similarly, a recent meta-analysis demonstrated that elevated CONUT scores were associated with poorer overall, disease-free, and relapse-free survival, supporting the importance of immunonutritional status in colorectal cancer prognosis [33,34].

It should also be emphasized that NLR, PLR, and PNI were assessed exclusively from preoperative laboratory measurements. Therefore, the present findings reflect baseline preoperative inflammatory and immunonutritional status and cannot provide information regarding dynamic perioperative changes in these biomarkers, which may have additional prognostic significance.

The stratified analysis according to surgical setting provided additional insight into these findings. As expected, emergency patients had significantly higher NLR and PLR values than elective patients, consistent with the influence of acute inflammatory stress associated with obstruction, perforation, infection, or other emergency presentations. Nevertheless, neither NLR nor PLR was independently associated with metastatic lymph node burden when the elective and emergency subgroups were analyzed separately, supporting the robustness of the negative findings for these inflammatory ratios. In contrast, the modest association between PNI and nodal burden was observed only in the emergency subgroup and not in elective patients. This subgroup difference suggests that the PNI finding should be interpreted cautiously and may partly reflect clinical heterogeneity related to emergency presentation rather than a uniform relationship with tumor biology.

Importantly, the modest association between PNI and metastatic lymph node burden observed in the primary analysis was attenuated after additional adjustment for total lymph node yield. Similar attenuation was observed for mucinous component and tumor location, whereas lymphovascular invasion remained independently associated with greater metastatic lymph node burden. Moreover, inclusion of PNI did not meaningfully improve overall model fit, suggesting limited incremental predictive value beyond the other clinical and pathological covariates. These findings highlight the importance of accounting for the extent of pathological nodal examination when metastatic lymph node count is analyzed as an outcome and suggest that some associations observed in the primary model may partly reflect differences in lymph node yield.

The additional subgroup and interaction analyses did not provide strong evidence that the associations of NLR, PLR, or PNI with metastatic lymph node burden differed according to tumor differentiation, mucinous status, surgical urgency, or tumor location. Although PNI was significantly associated with nodal burden in the G1–G2 and emergency subgroups, formal interaction testing did not confirm significant effect modification. In particular, the PNI-by-tumor-location interaction was not significant. These subgroup findings should therefore be regarded as exploratory.

Overall, PNI showed a modest association with metastatic lymph node burden in the primary model, but this association was not robust to additional adjustment for total lymph node yield and was inconsistent across subgroup analyses. Similarly, the association with tumor location was attenuated after adjustment for lymph node yield. In contrast, lymphovascular invasion remained independently associated with greater metastatic lymph node burden, supporting a more robust relationship between this pathological feature and the extent of nodal involvement.

Growing evidence indicates that conventional TNM staging alone does not fully explain outcome heterogeneity in colorectal cancer. Host-related factors, particularly nutritional and inflammatory status, provide complementary prognostic information beyond anatomical staging, and recent prognostic models increasingly combine immunonutritional biomarkers with clinicopathological variables to improve individualized risk prediction [2,3,22,35,36]. Consistent with this concept, emerging composite indices such as the CALLY index and the combined PIV–PNI score have demonstrated improved prognostic performance compared with isolated biomarkers, suggesting that multidimensional prediction models may represent the next generation of prognostic tools in colorectal cancer [10,11,37].

Together with previous evidence highlighting the importance of accurate lymph node assessment and pathological staging, our findings support the development of integrated prognostic models combining clinicopathological characteristics with readily available hematological biomarkers to improve prediction of metastatic lymph node burden [38,39,40,41].

Our findings are consistent with those of our previous cohort study, supporting the concept that integrating clinicopathological characteristics with routinely available biomarkers may improve prognostic assessment in colorectal cancer [40].

Furthermore, accumulating evidence indicates that the prognostic significance of PNI extends beyond patients undergoing curative surgery to those with metastatic disease receiving systemic therapy, emphasizing the importance of host immunonutritional status throughout the disease course [42].

### 4.1. Clinical Implications

The present findings have several potential clinical implications. The preoperative inflammatory and immunonutritional biomarkers evaluated in this study, including NLR, PLR, and PNI, are inexpensive, reproducible, and widely available parameters that can be derived from routine laboratory tests without additional costs or specialized investigations. Among these biomarkers, PNI retained a modest independent association with metastatic lymph node burden, whereas NLR and PLR did not. The surgical setting itself was not independently associated with metastatic lymph nodal burden after multivariable adjustment. However, because the association between PNI and nodal burden was confined to emergency patients in the stratified analysis, this finding should be interpreted cautiously and should not currently influence surgical or oncological decision-making. Nevertheless, PNI may warrant further investigation as a component of integrated clinicopathological prediction models. Accordingly, inflammatory and immunonutritional biomarkers should be interpreted together with tumor characteristics, radiological findings, biopsy features, and other established prognostic factors rather than used as isolated predictors [40,41].

PNI deserves particular attention because, unlike isolated inflammatory ratios, it reflects both nutritional and immunological status and therefore represents a potentially modifiable host-related parameter. Contemporary evidence supports routine nutritional screening, nutritional prehabilitation, oral nutritional supplementation, immunonutrition in selected patients, and multimodal perioperative nutritional optimization as important components of colorectal cancer care. These interventions may improve postoperative recovery, reduce complications, and increase treatment tolerance, emphasizing that impaired immunonutritional status represents an opportunity for targeted intervention rather than merely a prognostic indicator [17,18,42,43].

Malnutrition is common among patients undergoing colorectal cancer surgery and is associated with impaired immunity, infectious complications, anastomotic leakage, prolonged hospitalization, delayed recovery, and poorer oncological outcomes. The modest independent association observed between PNI and metastatic lymph node burden should therefore be interpreted cautiously and does not establish a causal relationship between nutritional status and lymphatic dissemination. Nevertheless, nutritional assessment should remain an integral component of routine perioperative management because of its established relevance to postoperative recovery and long-term outcomes [18,19,20,25,44,45,46].

Among the clinicopathological variables, tumor location and lymphovascular invasion were independently associated with metastatic lymph node burden, with lymphovascular invasion showing a particularly strong association with higher nodal burden. Perineural invasion also showed a borderline association with greater metastatic lymph node burden (adjusted IRR 1.844, 95% CI 0.987–3.443; *p* = 0.055), suggesting a potential relationship that warrants further investigation in larger, adequately powered cohorts. These findings reinforce the importance of tumor-related pathological characteristics in determining the extent of nodal involvement. Recognition of anatomical and pathological features associated with greater nodal burden may support careful preoperative staging, strict adherence to oncological surgical principles, and thorough pathological evaluation of the resected specimen. Nevertheless, neither tumor location nor the present exploratory associations should be used in isolation to determine the extent of surgery or lymphadenectomy; they should instead be interpreted within the broader context of imaging findings, pathological staging, molecular profiling, and established clinical criteria.

Finally, these findings are consistent with the movement toward personalized colorectal cancer management. Recent prognostic models integrating inflammatory and immunonutritional biomarkers with clinicopathological and molecular variables have demonstrated improved predictive performance compared with conventional TNM staging alone. The present results suggest that combining readily available host-related information such as PNI with tumor-related characteristics, particularly anatomical location and lymphovascular invasion, may warrant further investigation in future integrated prediction models of metastatic lymph node burden [1,3,5,12,33,47].

### 4.2. Novelty of the Study

The novelty of the present study should therefore be interpreted primarily from a clinical and methodological perspective rather than as evidence of a novel biological mechanism. By modeling the absolute number of metastatic lymph nodes as a quantitative count outcome, rather than considering nodal involvement only as a binary N0/N+ variable, the present analysis provides a more granular assessment of metastatic nodal burden. The findings further suggest that the contribution of routinely available immunonutritional biomarkers is limited compared with established tumor-related characteristics, although the modest association observed for PNI, particularly in the emergency setting, warrants further investigation in prospective cohorts.

A second strength is the use of multivariable negative binomial regression, an analytical approach specifically suited for overdispersed count data. This method allowed direct modeling of the number of metastatic lymph nodes and estimation of incidence rate ratios while simultaneously accounting for multiple clinicopathological and laboratory variables. In addition, the low variance inflation factors (VIF 1.01–1.17) confirmed the absence of relevant multicollinearity among the evaluated continuous predictors.

The study also benefits from a relatively large cohort of 935 patients with histologically confirmed colorectal adenocarcinoma. Notably, the fully adjusted analysis identified tumor location and lymphovascular invasion as independent tumor-related correlates of metastatic lymph node burden, while PNI showed a more modest independent association. In contrast, NLR and PLR were not independently associated with the outcome. These findings suggest that quantitative nodal burden may reflect an interplay between tumor-related characteristics and host immunonutritional status rather than being adequately characterized by inflammatory biomarkers alone.

Overall, this study demonstrates the value of combining routinely available laboratory biomarkers with clinicopathological variables within an appropriate count regression framework. By integrating anatomical location, pathological characteristics, and host-related biomarkers, the present approach provides a more comprehensive assessment of factors associated with metastatic lymph node burden than conventional binary analyses. However, the modest association observed for PNI warrants cautious interpretation and external validation. This methodological framework may serve as a basis for future prognostic models integrating clinical, pathological, molecular, and laboratory data [1,2,3,10,11,12,22,36].

### 4.3. Strengths and Limitations

The present study has several strengths. It included a relatively large cohort of 935 patients with histologically confirmed colorectal adenocarcinoma, with clinicopathological and laboratory variables obtained from routine clinical practice. The use of negative binomial regression, an appropriate statistical method for overdispersed count data, enabled quantitative evaluation of metastatic lymph node burden rather than conventional binary classification of nodal status. Furthermore, the fully adjusted multivariable model simultaneously evaluated routinely available inflammatory biomarkers (NLR and PLR), an immunonutritional biomarker (PNI), and relevant clinicopathological characteristics, including tumor location, tumor grading, mucinous component, lymphovascular invasion, and perineural invasion, allowing assessment of their independent associations with metastatic lymph node burden. Multicollinearity assessment demonstrated low variance inflation factors, indicating no relevant collinearity among the evaluated continuous predictors.

Another strength of the present study is the detailed evaluation of tumor location. Rather than comparing right- and left-sided colorectal cancer, we analyzed seven distinct anatomical locations, identifying significant location-specific differences in metastatic lymph node burden, with cecal tumors serving as the reference category. The inclusion of lymphovascular and perineural invasion in the expanded multivariable model also allowed adjustment for important pathological features related to tumor dissemination.

Nevertheless, several limitations should be acknowledged. First, the retrospective single-center design may have introduced selection bias and limits the generalizability of the findings. Because the excluded patients were not retained in a structured analytical dataset, the pattern of missingness by individual variable could not be reliably reconstructed, and a formal comparison of the characteristics of included and excluded patients could not be performed. Therefore, selection bias related to the complete-case approach cannot be excluded. In addition, no independent external cohort was available for validation. Therefore, the reproducibility of the observed associations across different institutions, patient populations, surgical practices, and pathological assessment protocols remains uncertain. The present findings should consequently be regarded as exploratory and hypothesis-generating until externally validated in independent multicenter cohorts.

Second, emergency presentations accounted for a relatively high proportion of the cohort (392/935, 41.9%), likely reflecting the case mix of our tertiary referral center. This center-specific characteristic may limit the generalizability of the findings to populations and institutions in which elective colorectal cancer surgery predominates. Emergency and elective patients differed significantly in several preoperative laboratory and clinicopathological characteristics, including NLR, PLR, hemoglobin, and PNI, confirming that acute clinical presentation may influence these biomarkers. To address this potential source of confounding, surgical setting was incorporated into the fully adjusted multivariable model, and additional stratified sensitivity analyses were performed. Surgical setting was not independently associated with metastatic lymph node burden in the overall model, and neither NLR nor PLR was independently associated with metastatic lymph node burden in either subgroup. However, residual confounding related to the severity and specific cause of emergency presentation, including obstruction, perforation, infection, or other acute inflammatory conditions, cannot be completely excluded. Moreover, the modest association observed for PNI was confined to the emergency subgroup and should therefore be interpreted cautiously.

Third, several potentially relevant variables, including body mass index, comorbidities, C-reactive protein, carcinoembryonic antigen, CA19-9, neoadjuvant treatment, nutritional interventions, tumor deposits, microsatellite instability, and molecular alterations such as *KRAS* and *BRAF*, were unavailable and therefore could not be incorporated into the multivariable analysis. Although the expanded model incorporated important pathological variables, including lymphovascular and perineural invasion, residual confounding from unavailable clinical, treatment-related, pathological, and molecular factors cannot be excluded. Consequently, the observed associations should be interpreted as exploratory rather than establishing causal or definitive predictive relationships.

The number of detected metastatic lymph nodes is influenced by total lymph node yield, and residual variability related to nodal assessment cannot be excluded despite adjustment for lymph node yield. A small number of potentially influential observations were identified but retained because there was no a priori justification for exclusion. Although the overall cohort was relatively large, only 215 patients had metastatic lymph nodes, and some degree of overfitting cannot be excluded. No formal internal validation using bootstrap resampling or cross-validation was performed; therefore, the apparent calibration estimates may be optimistic, and coefficient stability and optimism in model performance could not be formally assessed. Moreover, the model showed modest overall performance and PNI provided limited incremental predictive value. Consequently, the clinical applicability of these findings remains uncertain, and the results should be considered exploratory. Further validation in independent cohorts is required before any potential clinical implementation.

Furthermore, mucinous differentiation was recorded only as present or absent, without quantitative assessment of the proportion of extracellular mucin. Because the biological and metastatic behavior of colorectal tumors may vary according to the extent of mucinous differentiation, this dichotomous classification may have reduced pathological granularity and introduced residual heterogeneity within the mucinous group. Consequently, the independent association observed for the presence of a mucinous component should be interpreted cautiously and requires validation in studies incorporating standardized quantitative assessment of mucinous differentiation.

All inflammatory and immunonutritional biomarkers were assessed using a single preoperative measurement, and standardized serial postoperative measurements at 1, 3, and 6 months were not available because of the retrospective study design. This represents an important limitation of the present study. Consequently, the analysis reflects only baseline preoperative inflammatory and immunonutritional status and cannot determine whether longitudinal changes in NLR, PLR, or PNI provide additional prognostic information. Prospective studies incorporating standardized serial perioperative and postoperative biomarker assessments are warranted to address this issue. Likewise, long-term oncological outcomes, including recurrence, disease-free survival, overall survival, postoperative complications, and response to adjuvant therapy, were beyond the scope of the present study.

Consequently, prospective multicenter studies incorporating serial biomarker assessment, comprehensive molecular profiling, standardized pathological evaluation, and long-term follow-up are required to validate these findings. Particular attention should be given to confirming the independent associations of tumor location and lymphovascular invasion with metastatic lymph node burden and to determining whether the modest association observed for PNI remains significant after more comprehensive adjustment for clinical, pathological, treatment-related, and molecular factors.

### 4.4. Future Perspectives

Future prospective multicenter studies are needed to validate the present findings in independent populations and to determine whether the associations observed between routinely available inflammatory and immunonutritional biomarkers, including NLR, PLR, and PNI, and metastatic lymph node burden are reproducible across different clinical settings. Particular emphasis should be placed on confirming the independent association between tumor location and metastatic lymph node burden, while further clarifying the contribution of inflammatory and immunonutritional biomarkers after adjustment for established clinicopathological characteristics. Future investigations should also evaluate these biomarkers as dynamic parameters by assessing perioperative changes, including postoperative decline and subsequent recovery, because accumulating evidence suggests that longitudinal inflammatory and immunonutritional assessment provides greater prognostic information than a single preoperative measurement [1,16,24,25,48,49,50,51].

Future prognostic models should integrate inflammatory and immunonutritional biomarkers with established clinicopathological variables, detailed pathological characteristics, molecular alterations (including microsatellite instability, *KRAS*, *NRAS*, and *BRAF* mutations), radiomic features, and artificial intelligence-based imaging analysis to improve individualized prediction of metastatic lymph node burden and long-term oncological outcomes [49]. Comparative studies should also evaluate whether combining NLR, PLR, and PNI with other inflammatory and nutritional biomarkers, such as C-reactive protein, CONUT, SII, SIRI, CALLY, hemoglobin, red blood cell distribution width, and other composite indices, provides incremental predictive value beyond conventional clinicopathological models [40,50].

Future research should additionally investigate potential nonlinear relationships between inflammatory and immunonutritional biomarkers and metastatic lymph node burden using approaches such as restricted cubic splines or clinically relevant biomarker categories. Future prospective multicenter studies should incorporate serial biomarker assessment, comprehensive molecular profiling, standardized pathological evaluation, and long-term follow-up. Importantly, external validation in independent and geographically distinct multicenter cohorts will be required to assess the reproducibility and generalizability of the present findings across different clinical settings. Finally, any future integrated prediction model combining laboratory biomarkers with radiological, histological, molecular, and clinicopathological data should undergo rigorous internal and external validation, calibration assessment, and decision-curve analysis before implementation in routine clinical practice [1,3,5,10,11,12,22,34,46,48,52].

## 5. Conclusions

In this retrospective cohort, lymphovascular invasion showed the most robust association with metastatic lymph node burden and remained independently associated with greater nodal burden after adjustment for total lymph node yield. NLR and PLR were not independently associated with nodal burden, while PNI showed only a modest association in the primary model and provided no significant incremental improvement in model fit beyond the clinicopathological covariates. Associations involving tumor location were sensitive to model specification and should therefore be interpreted cautiously. Given the retrospective single-center design, potential residual confounding, and absence of external validation, these findings should be considered exploratory and require confirmation in prospective multicenter cohorts.

## Figures and Tables

**Table 1:** Baseline characteristics of the study population (*n* = 935).

Variable	Value
**Age, years**	67.9 ± 10.4
**Male sex**	539 (57.6)
**Hemoglobin, g/dL**	12.09 (10.30–13.10)
**Neutrophil-to-lymphocyte ratio (NLR)**	2.93 (2.34–4.12)
**Platelet-to-lymphocyte ratio (PLR)**	155.00 (118.34–221.71)
**Prognostic nutritional index (PNI)**	33.00 (26.00–40.83)
**Tumor location**	
**Cecum**	143 (15.3)
**Ascending colon**	123 (13.2)
**Hepatic flexure**	54 (5.8)
**Transverse colon**	73 (7.8)
**Splenic flexure**	66 (7.1)
**Descending colon**	75 (8.0)
**Sigmoid colon**	401 (42.9)
**Tumor grading**	
**G1**	66 (7.1)
**G1–G2**	70 (7.5)
**G2**	733 (78.4)
**G3**	66 (7.1)
**Mucinous component**	
**Absent**	732 (78.3)
**Present**	203 (21.7)

**Abbreviations**: **NLR**, neutrophil-to-lymphocyte ratio; **PLR**, platelet-to-lymphocyte ratio; **PNI**, prognostic nutritional index. Values are presented as mean ± SD, median (IQR), or *n* (%), as appropriate.

**Table 2:** Comparison according to lymph node metastasis status (*n* = 935).

Variable	N0 (*n* = 720)	N+ (*n* = 215)	*p*-Value
**Hemoglobin, g/dL**	12.10 (10.30–13.10)	11.90 (10.31–12.97)	0.575
**NLR**	2.93 (2.27–4.08)	2.93 (2.49–4.40)	0.404
**PLR**	155.15 (119.00–223.92)	145.45 (117.75–217.04)	0.364
**PNI**	33.00 (26.00–40.06)	34.00 (28.10–41.97)	0.111
**Tumor grading**	—	—	<0.001
**Tumor location**	—	—	0.696
**Mucinous component**	—	—	<0.001

**Abbreviations: N0**, no metastatic lymph nodes; **N+**, at least one metastatic lymph node; **NLR**, neutrophil-to-lymphocyte ratio; **PLR**, platelet-to-lymphocyte ratio; **PNI**, prognostic nutritional index. Values are presented as median (IQR). Continuous variables were compared using the Mann–Whitney U test, whereas categorical variables were compared using the chi-square test.

**Table 3:** Univariate negative binomial regression for the number of positive lymph nodes (*n* = 935).

Predictor	IRR	95% CI	*p*-Value
**Hemoglobin, per 1 g/dL increase**	0.971	0.934–1.009	0.129
**NLR, per 1-unit increase**	1.009	0.990–1.028	0.357
**PLR, per 10-unit increase**	0.999	0.998–1.000	0.220
**PNI, per 1-unit increase**	1.012	1.004–1.021	0.005
**Tumor grading, overall**	—	—	0.114
**G1**	**Reference**	—	—
**G1–G2**	1.506	0.956–2.371	0.077
**G2**	1.152	0.808–1.641	0.434
**G3**	1.373	0.863–2.184	0.181
**Tumor location, overall**	—	—	<0.001
**Cecum**	**Reference**	—	—
**Ascending colon**	0.715	0.509–1.005	0.054
**Hepatic flexure**	0.548	0.340–0.882	0.013
**Transverse colon**	0.878	0.596–1.295	0.512
**Splenic flexure**	0.859	0.574–1.286	0.461
**Descending colon**	1.709	1.201–2.434	0.003
**Sigmoid colon**	1.031	0.797–1.333	0.817
**Mucinous component**	—	—	<0.001
**Absent**	**Reference**	—	—
**Present**	2.011	1.657–2.442	<0.001

**Abbreviations:** IRR, incidence rate ratio; CI, confidence interval; NLR, neutrophil-to-lymphocyte ratio; PLR, platelet-to-lymphocyte ratio; PNI, prognostic nutritional index. An IRR > 1 indicates an increase, whereas an IRR < 1 indicates a decrease in the expected number of positive lymph nodes. Reference categories were G1 for tumor grading, cecum for tumor location, and absence of a mucinous component for mucinous status.

**Table 4:** Fully adjusted multivariable negative binomial regression for metastatic lymph node burden, including surgical setting (*n* = 935).

Predictor	Adjusted IRR	95% CI	*p*-Value
**Age, per 10-year increase**	0.835	0.683–1.022	0.080
**Male sex**	0.895	0.586–1.369	0.609
**Hemoglobin, per 1 g/dL increase**	0.966	0.872–1.071	0.513
**NLR, per 1-unit increase**	1.057	0.966–1.156	0.227
**PLR, per 10-unit increase**	0.995	0.966–1.024	0.733
**PNI, per 1-unit increase**	**1.024**	**1.001–1.047**	**0.045**
**Tumor grading, overall**	—	—	0.169
**G1**	Reference	—	—
**G1–G2**	0.511	0.202–1.293	0.156
**G2**	0.789	0.460–1.353	0.389
**G3**	1.405	0.635–3.108	0.402
**Tumor location, overall**	—	—	**0.021**
**Cecum**	Reference	—	—
**Ascending colon**	0.491	0.221–1.089	0.080
**Hepatic flexure**	0.560	0.219–1.433	0.226
**Transverse colon**	0.570	0.235–1.384	0.214
**Splenic flexure**	**0.380**	**0.153–0.940**	**0.036**
**Descending colon**	**0.165**	**0.066–0.414**	**<0.001**
**Sigmoid colon**	**0.527**	**0.281–0.990**	**0.047**
**Mucinous component, present vs. absent**	**1.768**	**1.072–2.915**	**0.026**
**Lymphovascular invasion, present vs. absent**	2.197	1.186–4.073	0.012
**Perineural invasion, present vs. absent**	1.844	0.987–3.443	0.055
**Surgical setting**	—	—	—
**Elective surgery**	Reference	—	—
**Emergency surgery**	0.939	0.608–1.450	0.776

**Abbreviations**: IRR, incidence rate ratio; CI, confidence interval; NLR, neutrophil-to-lymphocyte ratio; PLR, platelet-to-lymphocyte ratio; PNI, Prognostic Nutritional Index. **Note**: Adjusted IRRs were estimated using a fully adjusted multivariable negative binomial regression model including age, sex, hemoglobin, NLR, PLR, PNI, tumor grading, tumor location, mucinous component, lymphovascular invasion, perineural invasion, and surgical setting. Reference categories were female sex, G1 tumor grade, cecal tumor location, absence of mucinous component, absence of lymphovascular invasion, absence of perineural invasion, and elective surgery.

**Table 5:** Multicollinearity diagnostics for continuous laboratory and immunonutritional biomarkers included in the regression model.

Predictor	VIF
**Hemoglobin**	1.04
**NLR**	1.16
**PLR**	1.17
**PNI**	1.01

**Abbreviations**: VIF, variance inflation factor; NLR, neutrophil-to-lymphocyte ratio; PLR, platelet-to-lymphocyte ratio; PNI, prognostic nutritional index.

**Table 6:** Comparison of baseline characteristics according to surgical setting (*n* = 935).

Variable	Elective Surgery (*n* = 543)	Emergency Surgery (*n* = 392)	*p*-Value
**Age, years**	69 (61–74)	70 (62–76)	0.037
**Male sex, *n* (%)**	313 (57.6)	226 (57.7)	1.000
**Hemoglobin, g/dL**	12.22 (10.67–13.45)	11.90 (9.80–12.60)	**<0.001**
**NLR**	2.87 (2.04–3.57)	2.97 (2.51–5.39)	**<0.001**
**PLR**	145.00 (115.06–207.66)	172.06 (124.85–235.82)	**<0.001**
**PNI**	33.00 (26.00–43.00)	32.00 (26.00–37.02)	**0.002**
**Metastatic lymph nodes**	0 (0–0)	0 (0–1)	**0.004**
**Mucinous component, *n* (%)**	134 (24.7)	82 (20.9)	0.205
**Lymphovascular invasion, *n* (%)**	67 (12.3)	63 (16.1)	0.126
**Perineural invasion, *n* (%)**	69 (12.7)	74 (18.9)	**0.013**
**Tumor grading, overall**	—	—	**0.003**
**G1**	26 (4.8)	40 (10.2)	—
**G1–G2**	34 (6.3)	36 (9.2)	—
**G2**	445 (82.0)	288 (73.5)	—
**G3**	38 (7.0)	28 (7.1)	—
**Tumor location, overall**	—	—	**<0.001**
**Cecum**	64 (11.8)	79 (20.2)	—
**Ascending colon**	86 (15.8)	37 (9.4)	—
**Hepatic flexure**	36 (6.6)	18 (4.6)	—
**Transverse colon**	35 (6.4)	38 (9.7)	—
**Splenic flexure**	26 (4.8)	40 (10.2)	—
**Descending colon**	55 (10.1)	20 (5.1)	—
**Sigmoid colon**	241 (44.4)	160 (40.8)	—

**Abbreviations**: NLR, neutrophil-to-lymphocyte ratio; PLR, platelet-to-lymphocyte ratio; PNI, Prognostic Nutritional Index. Continuous variables are presented as median (IQR) and were compared using the Mann–Whitney U test; categorical variables were compared using the chi-square test.

**Table 7:** Sensitivity analysis of inflammatory and immunonutritional biomarkers according to surgical setting.

Predictor	Elective Adjusted IRR (95% CI)	*p*-Value	Emergency Adjusted IRR (95% CI)	*p*-Value
**Age, per 10 years**	1.149 (0.841–1.571)	0.382	0.855 (0.672–1.088)	0.202
**Male sex**	0.583 (0.310–1.098)	0.095	0.676 (0.386–1.182)	0.170
**Hemoglobin**	0.999 (0.835–1.195)	0.992	1.029 (0.914–1.159)	0.635
**NLR**	1.215 (0.961–1.536)	0.103	1.001 (0.937–1.070)	0.978
**PLR, per 10 units**	0.981 (0.927–1.038)	0.507	0.995 (0.966–1.026)	0.761
**PNI**	1.007 (0.976–1.040)	0.658	**1.036 (1.003–1.071)**	**0.034**

**Abbreviations**: IRR, incidence rate ratio; CI, confidence interval; NLR, neutrophil-to-lymphocyte ratio; PLR, platelet-to-lymphocyte ratio; PNI, Prognostic Nutritional Index. Models were adjusted for age, sex, hemoglobin, NLR, PLR, and PNI within each surgical-setting subgroup.

**Table 8:** Apparent (in-sample) performance and calibration assessment of the negative binomial regression model.

Performance Metric	Value
**McFadden pseudo-R^2^**	0.024
**Mean absolute error (MAE), metastatic lymph nodes**	1.807
**Root mean squared error (RMSE), metastatic lymph nodes**	3.732
**Calibration intercept**	≈0.000
**Calibration intercept, 95% CI**	−0.197–0.197
**Calibration slope**	1.000
**Calibration slope, 95% CI**	0.660–1.341
**Secondary AUC for N+ vs. N0**	0.662
**Secondary Brier score for N+ vs. N0**	0.168

**Abbreviations**: AUC, area under the curve; N+, presence of at least one metastatic lymph node; N0, absence of metastatic lymph nodes. AUC and Brier score were calculated as secondary performance measures for discrimination of N+ versus N0 using probabilities derived from the fitted negative binomial model. The primary outcome remained the number of metastatic lymph nodes. Calibration estimates represent apparent (in-sample) calibration because they were obtained using the same dataset employed for model development. The calibration intercept and slope were estimated using negative binomial recalibration models, with model-based 95% confidence intervals derived from the corresponding regression standard errors. No bootstrap resampling or cross-validation was performed; therefore, these estimates were not corrected for optimism, and the near-ideal point estimates should not be interpreted as evidence of internal validation or validated calibration.

**Table 9:** Stratified negative binomial regression analyses of inflammatory and immunonutritional biomarkers according to tumor differentiation grade, mucinous status, and surgical setting.

Subgroup	*n*	N+	NLR, Adjusted IRR (95% CI)	*p*-Value	PLR, Per 10-Unit Increase, Adjusted IRR (95% CI)	*p*-Value	PNI, Adjusted IRR (95% CI)	*p*-Value
**Tumor differentiation grade**								
**G1**	66	21	0.744 (0.439–1.262)	0.273	0.924 (0.808–1.055)	0.243	1.038 (0.956–1.126)	0.375
**G1–G2**	70	17	0.670 (0.306–1.465)	0.315	1.056 (0.902–1.235)	0.499	**1.138 (1.022–1.268)**	**0.018**
**G2**	733	161	1.025 (0.928–1.132)	0.627	1.001 (0.968–1.035)	0.957	1.014 (0.987–1.042)	0.323
**G3**	66	16	1.243 (0.840–1.839)	0.276	0.944 (0.816–1.092)	0.436	0.941 (0.870–1.019)	0.134
**Mucinous status**								
**Absent**	732	146	1.068 (0.954–1.194)	0.254	1.004 (0.970–1.040)	0.807	1.012 (0.982–1.042)	0.443
**Present**	203	69	0.976 (0.870–1.094)	0.671	0.971 (0.923–1.022)	0.262	1.023 (0.986–1.061)	0.221
**Surgical setting**								
**Elective**	543	105	1.215 (0.961–1.536)	0.103	0.981 (0.927–1.038)	0.507	1.007 (0.976–1.040)	0.658
**Emergency**	392	110	1.001 (0.937–1.070)	0.978	0.995 (0.966–1.026)	0.761	**1.036 (1.003–1.071)**	**0.034**

**Abbreviations**: CI, confidence interval; IRR, incidence rate ratio; N+, patients with at least one metastatic lymph node; NLR, neutrophil-to-lymphocyte ratio; PLR, platelet-to-lymphocyte ratio; PNI, prognostic nutritional index. Note: Separate negative binomial regression models were fitted within each subgroup, with metastatic lymph node count as the dependent variable. Models were adjusted for age, sex, hemoglobin, NLR, PLR, and PNI. PLR was modeled per 10-unit increase. Statistically significant associations (*p* < 0.05) are shown in bold. Formal interaction testing showed no statistically significant effect modification by tumor differentiation grade, mucinous status, surgical setting, or tumor location. The PNI-by-tumor-location interaction was not significant (overall *p* = 0.598), while the PNI-by-grade interaction approached statistical significance (*p* = 0.055).

## Data Availability

The data presented in this study are available on request from the corresponding author. The data are not publicly available due to patient confidentiality.
